# Augmented reality guided versus computed tomography guided percutaneous lung nodule localization: a noninferiority randomized clinical trial

**DOI:** 10.1097/JS9.0000000000002308

**Published:** 2025-02-24

**Authors:** Zuodong Song, Yin Li, Qing Tian, Chao Sun, Hongfeng Liu, Kongyong Chong, Qian Zhang, Jianeng Chen, Pengchong Li, Liwei Song, Davide Tosi, Min P. Kim, Zhebing Lin, Qingquan Luo, Lingming Yu, Xinghua Cheng

**Affiliations:** a Department of Oncology, Shanghai Lung Cancer Center, Shanghai Chest Hospital, Shanghai Jiao Tong University School of Medicine, Shanghai, China; b Department of Thoracic Surgery, the First Hospital of Hebei Medical University, Hebei, China; c Department of Thoracic Surgery, Donghai County People’s Hospital, Jiangsu, China; d Department of Oncology, Shandong Provincial Hospital, Shandong University, Shandong, China; e Department of Thoracic Surgery, Jining No. 1 People’s Hospital, Shandong, China; f Department of Thoracic Surgery, Hospital Kuala Lumpur, Malaysia; g Department of Thoracic Surgery, Zhangjiagang Hospital of Traditional Chinese Medicine, Nanjing University of Chinese Medicine, Jiangsu, China; h Thoracic Surgery and Lung Transplantation Unit, Fondazione IRCCS Ca’ Granda Ospedale Maggiore Policlinico, University of Milan, Milan, Italy; i Division of Thoracic Surgery, Department of Surgery, Houston Methodist Hospital, Houston, TX, USA,; j Department of Radiology, Shanghai Chest Hospital, Shanghai Jiao Tong University School of Medicine, Shanghai, China

**Keywords:** augmented reality, lung nodule localization, percutaneous transthoracic lung puncture, surgery

## Abstract

**Hypothesis:**

This study hypothesized that augmented reality (AR) technology has comparable accuracy and safety to conventional CT localization in guiding percutaneous transthoracic lung puncture (PTLP) to localize small pulmonary nodules.

**Methods:**

This study was a prospective, non-inferiority randomized clinical trial. Patients were randomly assigned between 23 May 2023, and 26 September 2023. Patients with small peripheral lung nodules (≤2 cm) were recruited. Patients were randomly assigned to either the CT-guided PTLP group or the AR-guided PTLP group, with a 1:1 allocation ratio. The primary outcome was the accuracy of lung nodule localization measured by localization error. The secondary outcomes included procedure duration, radiation exposure dosage and complications.

**Results:**

A total of 70 patients underwent either CT- or AR-guided lung nodule localization and subsequent surgeries. Localization error was smaller in the AR-guided group than in the CT-guided group (mean ± SD, 3.1 ± 4.0 mm vs. 5.4 ± 4.2 mm, *P* = 0.026). The mean difference of localization errors was −2.3 mm (95% CI: − 4.2 to −0.3 mm, *P* < 0.001 for non-inferiority). Compared to the CT-guided group, the AR-guided group demonstrated significantly lower radiation exposure (mean ± SD, 421 ± 168 vs. 694 ± 229 mGy × cm, *P <* 0.001) and shorter localization procedure duration (mean ± SD, 8.8 ± 2.3 vs. 14.1 ± 1.8 minutes, *P <* 0.001), with no statistical difference in complications.

**Conclusions:**

The accuracy of the AR-guided approach is comparable to that of the CT-guided approach in localizing small lung nodules. Furthermore, the utilization of AR technology has been demonstrated to reduce procedural time and minimize radiation exposure for patients.

## Introduction

Lung cancer is the leading cause of cancer-related death worldwide[1]. Computed tomography (CT)-guided percutaneous transthoracic lung puncture (PTLP) is an effective approach for diagnosing lung cancer and facilitating interventional treatment[2]. With the increasing detection of early-stage lung cancer through CT screening, preoperative localization of pulmonary nodules has become a critical step in ensuring precise sublobar resection for small lung cancer^[3,4]^. When considering factors such as maneuverability, accessibility, and cost, CT-guided PTLP is a widely utilized approach for this purpose^[5,6]^. However, this method generally requires coordination for same-day interventional radiology and operating room scheduling, and patients often experience prolonged pain and increased risk of undetected complications while waiting for surgery[7].

Augmented reality (AR), an innovative imaging technology that merges digital data with the real environment, has recently been incorporated into the development of surgical navigation systems. AR technology has been applied in various disciplines such as orthopedics and neurosurgery, to assist surgeons in preoperative planning and intraoperative navigation^[8-10]^. Our preliminary studies validating the potential of AR-guided PTLP for lung nodule localization have shown promising results in animal models[11]. To further evaluate its accuracy in human patients and explore its potential to replace CT-guided PTLP in localizing lung nodules, we initiated this clinical trial. In this trial, we assessed the accuracy of the two procedures and we investigated other aspects, including procedural time, radiation exposure, procedural time, and PTLP-related complications, as well as patient pain and stress level.

## Methods

### Study design

This was a prospective, single-institutional, non-inferiority randomized clinical trial that compared the AR-guided method to the conventional CT-guided approach for localizing small lung nodules. The study protocol is available at Clinicaltrials.gov. The primary objective of the study was to evaluate whether the AR-guided approach could achieve comparable precision in nodule localization. This work has been reported in line with the CONSORT criteria[12].

### Inclusion and exclusion criteria

Patients with lung nodules scheduled for sublobar resection via video-assisted thoracoscopic surgery (VATS) in our hospital were evaluated for eligibility by at least two surgeons. The main inclusion criteria were that patients aged ≥18 years, with no distant metastasis on preoperative evaluation, requiring lung nodule localization and sublobar resection, with nodule diameter ≤2 cm and at least 2 cm from the pulmonary artery or vein, ECOG score of 0/1, and signed informed consent. The main exclusion criteria were the presence of two or more lung nodules that required simultaneous resection, nodule located in scapular region obstructing percutaneous localization, uncontrollable mental illness, severe preoperative complications, need for emergency surgery, or voluntary withdrawal from the study.

### Intervention

#### Test arm

**Figure 1.**
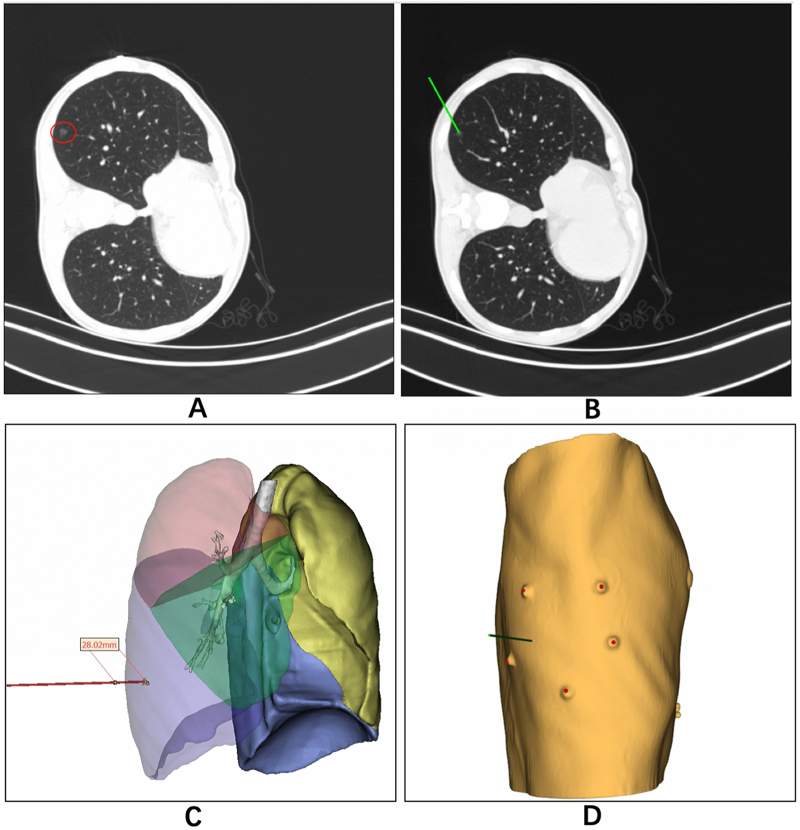
Preoperative CT and 3D planned path for the AR-guided PTLP for localizing lung nodule. The location of nodule (marked by the red cycle) was confirmed on the initial CT scan (A). Next, we planned the path for PTLP in the reconstruction software (the green line) (B), and a 3D digital twin model of the patient’s thorax with puncture path and depth was subsequently constructed (C). A model of five asymmetric labels on the chest wall for model registration and puncture guidance was also constructed (D).

**Figure 2.**
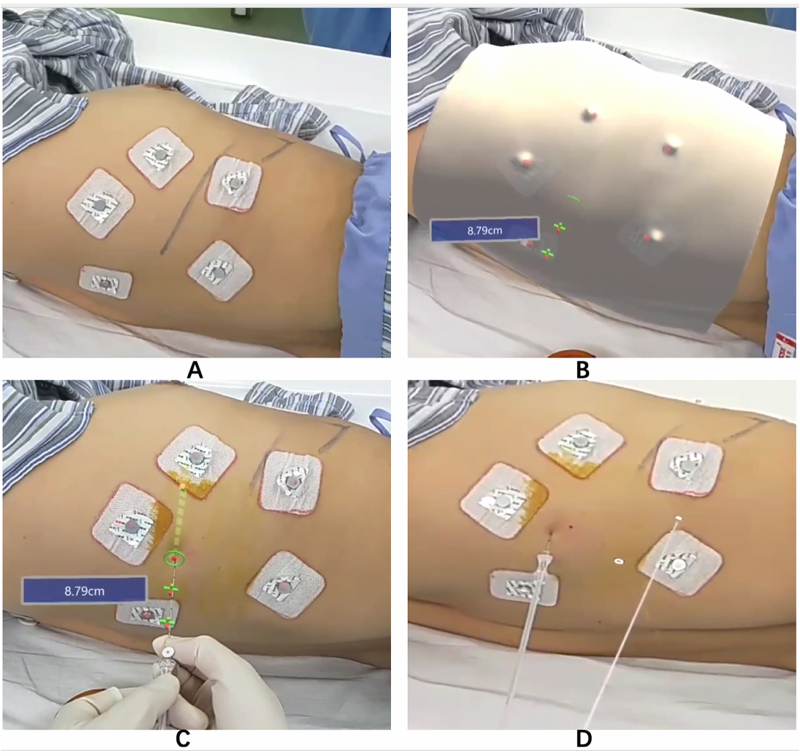
Actual scene images of the AR-guided PTLP procedure. The head-mounted AR system recognizes the labels on the patient’s chest wall (A) and matches the digital twin model to the patient’s actual thorax to display the virtual puncture path (B). The operator inserted the introducing needle at the virtual insertion point (the green ring with a central red dot) and placed the needle and the hookwire along the virtual path (two green crosses) to the predetermined depth (C, D).

PTLP was guided by a head-mounted AR system (JinJing system, XenovaMed, Shanghai, China) implemented in a HoloLens headset (Microsoft, Redmond, USA). In the test arm, the positioning of the CT scan upon admission was determined based on the requirements of the PTLP procedure. Prior to the CT scan, the patient had been fitted with five asymmetric labels affixed to the patient’s surgical side of the chest. These labels were designed to be uniquely identifiable by the AR headset. Alternatively, if PTLP could be performed in a supine position, these labels were placed on five fixed anatomical chest landmarks, including the upper edge of the sternum, left and right nipples, midpoint between the nipples, and lower end of the xiphoid process. Digital imaging and communications (DICOM) image data obtained from the CT scan upon admission (Fig. 1A) were processed using reconstruction software (JinJing system, XenovaMed, Shanghai, China) to plan the PTLP path and generate a three-dimensional (3D) digital twin model of the patient’s thorax with the virtual path (Fig. 1B–D). The model was then imported into 3D graphic software (Unity Technologies, San Francisco, USA) to create a suitable AR scene, and subsequently deployed into the HoloLens for AR-guided PTLP. For lung nodule localization, the surgeon who would perform pneumonectomy placed a needle following the virtual pathway that was visualized using the AR headset in the CT room (Fig. 2). The AR navigational system automatically matched the digital twin model of the patient’s thorax to the corresponding position of the patient’s actual thorax by identifying the labels on the patient’s chest wall (Fig. 2A, B). The pre-planned virtual puncture pathway insertion site, and puncture depth on the patients’ thorax was presented to the operator through the HoloLens headset, enabling the placement of the introducer needle under local anesthesia. Subsequent to the deployment of the hook wire through the needle, a CT scan was performed to assess the accuracy of the localization. Both the initial CT scan at admission and AR-guided PTLP were performed at the end of the patient’s normal inspiratory phase. After nodule localization, the patient was sent to the operating room for lung resection using VATS.

#### Control arm

In the CT-guided group, patients were positioned according to the requirements of the PTLP, and an initial CT scan was conducted in the area of interest. The insertion site for the introducing needle was determined using CT gantry laser light and a metal marker on the skin. The insertion length and angle were estimated based on the initial CT images obtained by radiologists. The PTLP in the CT-guided group was performed by radiologists, who utilized the same needle and hookwire as in the AR-guided group. Subsequently, the introducing needle was inserted halfway without penetrating the pleura under local anesthesia. Subsequent CT scans were performed to confirm or adjust the needle position to achieve accurate nodule localization. It was considered acceptable for satisfactory nodule localization when there was a distance of ≤2 cm between the puncture needle and the target nodule^[7,13]^. Once the relative position of the puncture needle was confirmed, a hookwire was placed through the introduction of needle, and another CT scan was immediately performed to assess the accuracy of the localization before the patient was sent for surgery.

### Primary outcomes

The primary outcome of this study was localization error, which was assessed by measuring the shortest distance between the hookwire and the closest edge of the target nodule based on the final CT scan immediately after the hookwire was placed. If the hookwire hits the nodules, the localization error is set to 0. Given that the hookwire and the target nodule may not be located on the same plane in a 2D CT scan, the localization error was measured by reconstructing the target nodule and the hookwire into 3D formats and measuring their 3D spatial distance using computer-aided design software (Mimics, Materialize, USA) (Supplemental Figure 1, available at: http://links.lww.com/JS9/D961).

### Secondary outcomes

The secondary outcomes included the duration of the localization procedure, the radiation exposure dose, the localization-related complications’ rate (e.g., pneumothorax, hemorrhage, and hemoptysis, as defined in Supplemental Methods 2, available at: http://links.lww.com/JS9/D961), and pain scores, and stress levels. The duration of the CT-guided localization procedure was determined by the time between the initial and final CT scans, whereas the AR-guided localization procedure was automatically recorded by the AR system from model matching to the final CT confirmation. The radiation dose received by patients (comprising the initial, during localization and final CT scans) was quantified using the dose-length product (DLP)[14]. The pain scores (0-10) at the puncture site were measured using the Numerical Rating Scale (NRS) (Supplemental Table 1, available at: http://links.lww.com/JS9/D961) and assessed by nurses who were blinded to the localization method immediately after puncture and on the first day after surgery. Patients’ stress levels were assessed using the Post-Traumatic Stress Disorder Checklist-Civilian Version (PCL-C) scale (Supplemental Table 2, available at: http://links.lww.com/JS9/D961) on the first postoperative day.

### Randomization

A computerized randomization list was created using block randomization, with a block size of 6. The results were placed in sealed envelopes by individuals who were not included in the study. Participants were assigned a random number upon providing written informed consent, based on the sequence of their enrollment. Owing to the unfeasibility of masking, this study was not blinded to the operator.

### Noninferiority margin and sample size

Based on the findings of our previous study[11] and others[15], we anticipated an expected difference in localization error between the CT- and AR-guided groups of 0.86 mm. The standard error for the test arm was 5.07 mm[11], while that for the control arm it was 5.80 mm[15]. To achieve 80% statistical power with a one-sided α of 0.025 and a non-inferiority margin of 5 mm, 66 patients (33 in each group) were randomized. To account for potential dropouts, we included 35 patients in each group.

### Statistical analysis

Analysis was by intention to treat. Statistical analysis was performed using SPSS 26.0 (IBM Corp., Armonk, NY, USA) and R (Vienna, Austria, version 3.6.2). Data from each group are presented as the mean ± standard deviation (SD) or median with interquartile range (IQR). We employed three types of statistical tests to determine whether the differences between the two groups were statistically significant: independent-sample *t*-tests for continuous variables that followed a normal distribution, χ2 tests for categorical variables, and Mann–Whitney U tests for continuous variables that did not conform to a normal distribution. The confidence interval method was used to evaluate whether the detection capability of the AR-guided method was within the boundary of the non-inferiority effect. Multiple logistic regression analysis was conducted and the localization error was categorized as a binary variable with a threshold of 10 mm. It is worth noting that owing to the presence of premature randomization, all statistical analyses were two-sided, and statistical significancewas defined as *P* <0.05. The cumulative sum (CUSUM) method was used to quantitatively assess the learning curve. CUSUM for operation time was calculated as follows: $CUSUM=\sum_{i=0}^{n}\left(x_{i}-u\right)$, where *x*_i_ and *u* respectively represent an individual and the mean overall operative time^[16,17]^. In addition, we established a polynomial trend line to show the change in the slope of the learning curve which was divided into three stages: ascending phase (Phase I), transition phase (Phase II), and maturity phase (Phase III).

## Results

**Figure 3.**
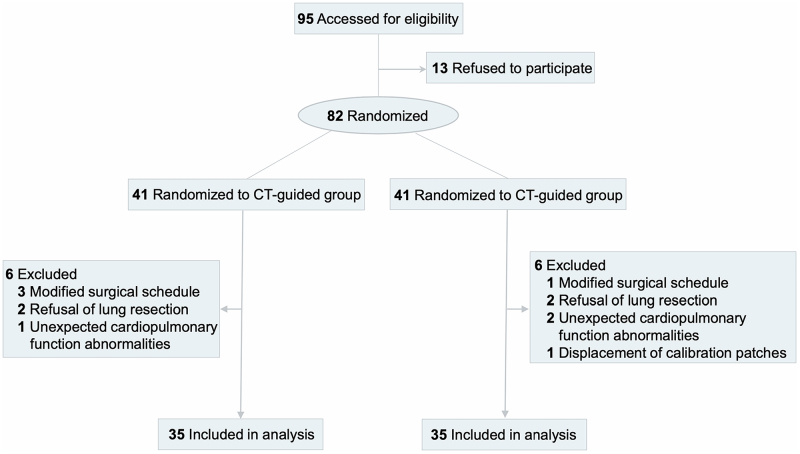
CONSORT Flowchart.

Between 23 May 2023, and 26 September 2023, a total of 95 patients underwent screening for eligibility, with 82 randomized to the CT- and AR-guided groups in a 1:1 ratio. Twelve patients were excluded from the intention-to-treat (ITT) analysis. Reasons for exclusion included modifications to surgical schedules (n = 4), patient refusal to undergo lung resection (n = 4), unexpected cardiopulmonary function abnormalities (n = 3), and displacement of labels (n = 1). A flowchart depicting participant enrollment is presented in the CONSORT Flow Diagram (Figure 3). Ultimately, 70 patients (35 in each group) were included in the final analysis. The clinical characteristics of the patients in both groups are summarized in Table 1.

**Table 1:** Clinical characteristics of the CT- and AR-guided group

Parameters	Total (n = 70)	CT-guided group (n = 35)	AR-guided group (n = 35)	*P*
Gender, n (%)				
Male	29 (41.4%)	15 (42.9%)	14 (41.2%)	0.127
Female	41 (58.6%)	20 (57.1%)	21 (58.8%)	
Age, year				0.750
Mean ± SD	57.5 ± 10.6	57.0 ± 10.2	57.97 ± 10.0	
BMI				0.215
Mean ± SD	22.6 ± 4.1	22.0 ± 5.1	23.2 ± 2.7	
Smoking history, n (%)				0.743
Yes	11 (15.7%)	6 (17.1%)	5 (14.3%)	
No	59 (84.3%)	29 (82.9%)	30 (85.7%)	
Nodule location, n (%)				0.302
RUL	24 (34.3%)	11 (31.4%)	13 (37.1%)	
RML	5 (7.1%)	4 (11.4%)	1 (2.9%)	
RLL	13 (15.6%)	8 (22.9%)	5 (14.3%)	
LUL	18 (25.7%)	6 (17.1%)	12 (34.3%)	
LLL	10 (14.3%)	6 (17.1%)	4 (11.4%)	
Nodular diameter, mm				0.180
Mean ± SD	10.4 ± 4.3	9.7 ± 3.7	11.1 ± 4.8	
Nodule type, n (%)				0.104
Pure GGO	32 (45.7%)	12 (34.3%)	20 (57.1%)	
Mixed GGO	30 (42.9%)	17 (48.6%)	13 (37.1%)	
Solid	8 (11.4%)	6 (17.1%)	2 (5.7%)	
Puncture depth, mm				0.124
Mean ± SD	64.7 ± 18.9	68.2 ± 16.7	61.2 ± 20.5	
Distance from pleura, mm				0.551
Mean ± SD	11.7 ± 10.4	11.0 ± 9.4	12.5 ± 11.4	
Patient position				0.578
Lateral position	53 (75.7%)	28 (80.0%)	25 (71.4%)	
Supine position	17 (24.3%)	7 (20.0%)	10 (28.6%)	
FEV1 (% predicted)				0.717
Mean ± SD	94.6 ± 15.9	95.3 ± 12.9	93.9 ± 18.6	
Operation type, n (%)				0.811
Wedge resection	37 (52.9%)	19 (54.3%)	18 (51.4%)	
Segment resection	33 (47.1%)	16 (45.7%)	17 (48.6%)	
Margin distance, mm				0.325
Mean ± SD	18.0 ± 8.4	17.0 ± 7.5	19.0 ± 9.2	
Frozen section results, n (%)				0.322
Benign	8 (11.4%)	5 (14.3%)	3 (8.6%)	
AAH	3 (4.3%)	2 (5.7%)	1 (2.9%)	
AIS	10 (14.3%)	3 (8.6%)	7 (20.0%)	
MIA	40 (57.1%)	22 (62.9%)	18 (51.4%)	
IA	8 (11.4%)	2 (5.7%)	6 (17.1%)	
MT	1 (1.4%)	1 (2.9%)	0 (0)	

Abbreviations: ITT, intention to treat; AR, augmented reality; AAH, atypical adenomatous hyperplasia; AIS, adenocarcinoma in situ; BMI, body mass index; CT, computed tomography; FEV1, forced expiratory volume in 1 s; GGO, ground glass opacity; IA, invasive adenocarcinoma; LLL, left lower lobe; LUL, left upper lobe; MIA, minimally invasive adenocarcinoma; MT, metastatic tumor; RLL, right lower lobe; RML, right middle lobe; RUL, right upper lobe.

### Analysis of primary outcomes

Localization error was smaller for patients in the AR-guided group compared to that in the CT-guided group (mean ± SD, 3.1 ± 4.0 vs. 5.4 ± 4.2 mm; *P* = 0.026; Table 2). The mean difference in localization errors between the two groups was −2.3 mm (95% CI: − 4.2 mm to −0.3 mm, *P* < 0.001 for non-inferiority), which did not reach the predefined non-inferiority margin of 5 mm. The localization errors in both groups were within 2 cm (Supplemental Figure 2, available at: http://links.lww.com/JS9/D961). The absolute number of cases with a localization error ≤ 5 mm was larger in the AR-guided group (n = 25, 71.4%) than in the CT-guided group (n = 20, 57.1%), but the difference was not significant (*P* = 0.512; Supplemental Table 3, available at: http://links.lww.com/JS9/D961). In the AR-guided group, there was no statistical difference in localization error between patients with labels on anatomical chest landmarks (n = 10) and those with operator-selected asymmetric labels on the chest wall (n = 25) (2.7 ± 5.2 mm vs. 3.3 ± 3.5 mm; *P =* 0.711). In the multiple regression analysis for localization error within 10 mm, only puncture depth was significant (*P* = 0.046). The puncture guidance method, body mass index (BMI), nodule location, nodule diameter, and patient position did not appear to correlate with localization error (Table 3).

**Table 2:** Primary and secondary outcomes of the CT- and AR-guided group

	Total (n = 70)	CT-guided group (n = 35)	AR-guided group (n = 35)	*P*
Localization error, mm				0.026
Mean ± SD	4.3 ± 4.2	5.4 ± 4.2	3.1 ± 4.0	
Complications after puncture				
Pneumothorax, n (%)	4 (5.7%)	2 (5.7%)	2 (5.7%)	1
Hemoptysis, n	0	0	0	-
Hemorrhage, n	0	0	0	-
Radiation exposure dose, mGy × cm				<0.001
Mean ± SD	583 ± 254	694 ± 229	421 ± 168	
Localization procedure duration, min				<0.001
Mean ± SD	11.5 ± 3.4	14.1 ± 1.8	8.8 ± 2.4	
Pain score				
During puncture (median, IQR)	5 (4-6)	5 (3-6)	5 (4-6)	0.204
First day after surgery (median, IQR)	3 (2-4)	2 (2-4)	3 (2-6)	0.783
PCL-C score (median, IQR)	21 (18-28)	20 (18-23)	23 (18-29)	0.149

Abbreviations: AR, augmented reality; CT, computed tomography; PCL-C, Post-Traumatic Stress Disorder Checklist-Civilian Version.

**Table 3:** Multiple logistic regression analysis results of the factors affecting localization error within 10 mm

Variable		*P*
Guidance method, n (%)		0.898
CT-guided	35 (50%)	
AR-guided	35 (50%)	
BMI		0.120
Mean ± SD	22.6 ± 4.1	
Nodule location, n (%)		0.596
UL + ML	47 (67.1%)	
LL	23 (32.9%)	
Nodular diameter, mm		0.537
Mean ± SD	10.4 ± 4.3	
Puncture depth, mm		0.046
Mean ± SD	64.7 ± 18.9	
Patient position, n (%)		0.900
Lateral position	53 (75.7%)	
Supine position	17 (24.3%)	
Distance from pleura, mm		0.427
Mean ± SD	11.7 ± 10.4	

Abbreviations: AR, augmented reality; BMI, body mass index; CT, computed tomography; LL, lower lobe; ML, middle lobe; UL, upper lobe.

### Analysis of secondary outcomes

The radiation exposure dose was significantly lower in the AR-guided group than in the CT-guided group (mean ± SD, 421 ± 168 vs. 694 ± 229 mGy × cm, *P <* 0.001) (Table 2). The duration of the localization procedure was also significantly shorter in the AR-guided group than in the CT-guided group (mean ± SD, 8.8 ± 2.3 vs. 14.1 ± 1.8 min, *P <* 0.001) (Table 2). Regarding the complications associated with PTLP, there was no statistical difference in the occurrence of pneumothorax (6% vs. 6%), hemoptysis (0% vs. 0%), or hemorrhage (0% vs. 0%) between the two groups. Pain scores did not differ significantly between the CT- and AR-guided groups during the puncture procedure (median, 5 vs. 5, *P =* 0.204) or on the first day after surgery (median, 2 vs. 3, *P =* 0.783) (Table 2). Additionally, there was no significant difference in the patients’ stress levels on the first day after surgery between the CT- and AR-guided groups (median, 20 vs. 23, *P =* 0.149).

### Surgical and pathological outcomes

All resections were successful without positive margins, with 52.9% of patients undergoing wedge resections and the rest undergoing segmentectomies. The surgical outcomes and pathological results are summarized in Table 1. There was no significant difference in margin distance between the CT- and AR-guided groups (17.0 ± 7.5 mm vs. 19.0 ± 9.2 mm, *P =* 0.325). Intra-operative frozen section analysis revealed various subtypes of lung cancer and benign lesions. Following the frozen section analysis, eight patients diagnosed with invasive adenocarcinoma underwent lobectomy with lymph node dissection. No severe surgical morbidity or mortality was observed after surgery.

### Learning curve for AR-guided approach

Learning curve analysis for AR-guided localization was conducted considering the operator’s lack of prior PTLP experience (Supplemental Figure 3, available at: http://links.lww.com/JS9/D961). Three distinct phases were observed: Phase I (1–15 cases) represented the initial learning period. Phase II (15–20 cases) showed further improvement in surgical skills and Phase III (20–35 cases) indicated the achievement of procedural proficiency.

## Discussion

CT-guided PTLP is a commonly used technique for localizing lung nodules before sublobar resection. However, it is associated with increased patient discomfort and radiation exposure, additional clinician workload, and logistical burden for hospitals^[5-7]^. Consequently, there is a pressing need for novel techniques that can enhance procedural efficiency and reduce reliance on CT guidance, while maintaining localization accuracy.

In the present study, we compared the accuracy of a novel AR-guided PTLP[11] for localizing lung nodules using the conventional CT-guided approach. The average localization error in the AR group was smaller than in the CT group, and the difference being −2.3 mm (95%CI, − 4.2 to −0.3 mm; *P* < 0.001 for non-inferiority). The rates of localization errors within 5 mm, between 5 and 10 mm, and within 2 cm were also comparable between the two groups (Supplemental Figure 2 and Supplemental Table 3, available at: http://links.lww.com/JS9/D961). All patients in both groups underwent margin-negative resections, with no statistical difference in the average margin distance. Multiple regression analysis also showed no association between puncture guidance method and localization error. Currently, innovative lung nodule localization techniques are emerging, such as the use of claw suture devices or laser guidance. Similar to our findings, these methods have reported accuracy and safety comparable to CT localization. However, we believe that AR-guided localization can further reduce the reliance on CT for lung nodule localization by eliminating the need to repeatedly verify the position of the localization needle during the procedure.

For the measurement of localization error, we chose the distance between the hookwire and the closest edge of the target nodule rather than the nodule center for several reasons. First, many nodules have irregular and asymmetrical shapes, making it challenging to accurately define the central point, which could introduce bias. Second, it is not essential to place the hookwire precisely at the center for localization purposes^[13,15,18]^, and in some cases, navigating to the center may be hindered by the presence of ribs. In addition, the average tumor diameter was not significantly different between the CT and AR-guided groups (9.7 ± 3.7 vs. 11.1 ± 4.8 mm; *P* = 0.180), which should have limited impact on the results. The actual hit rates were 2.9% and 37.1% (Supplemental Table 3, available at: http://links.lww.com/JS9/D961) in the CT and AR groups respectively. Nevertheless, the distance between the hookwire and estimated tumor center was also measured (Supplemental Methods 1 and Supplemental Figure 4, available at: http://links.lww.com/JS9/D961), and no significant differences were observed between the two groups (Supplemental Figure 5 and Supplemental Table 4, available at: http://links.lww.com/JS9/D961). However, further investigation is needed to determine whether the implementation of AR could enhance the outcomes of other interventional procedures, such as biopsy and ablation.

The AR-guided approach also demonstrated shorter procedural durations, reduced radiation exposure, and similar complication rates when compared to the CT-guided approach, despite the operator’s lack of prior PTLP experience. This finding suggests that the implementation of AR has led to enhanced procedural efficiency. Notably, the operator achieved proficiency in the AR approach after only 20 cases, indicating a relatively short learning curve^[13,19]^. In this study, all PTLPs were conducted under local anesthesia, with no statistical differences in the patients’ pain and stress levels between the two groups. A subsequent multicenter randomized controlled study is currently underway to ascertain whether the implementation of an AR-guided approach in patients undergoing general anesthesia in the operating room would result in a further reduction in patient discomfort without compromising the successful resection rate.

Digital technologies, including augmented reality, hold the promise of data-driven precision surgery, ultimately aiming to improve patient outcomes, operative performance, and productivity and efficiency of surgeons and their teams^[20,21]^. AR has already been application in various disciplines such as orthopedics and neurosurgery^[8,10,22]^, assisting surgeons in both preoperative planning and intraoperative navigation. A comparison of AR localization technology with other intraoperative lung nodule localization technologies, such as magnetic navigation, robotic technology, and 3D printed navigational templates, demonstrates comparable accurac^[8,15,23-25]^. However, the superior accuracy of magnetic navigation and robotics is offset by their high costs, prolonged procedural durations, and stringent environmental requirements^[23,26-28]^. A similar observation can be made in the context of 3D printed navigational templates, which are characterized by elevated guidance expenses, the requirement of dedicated printing equipment and materials, lack of adaptability to patient alterations, and susceptibility to localization deviations due to minor positional shifts^[15,18]^. AR technology, in comparison with these other technologies, may have more developmental potential.

The present study is not without its limitations. First, the relatively small sample size and single-center design necessitate further validation in larger and more diverse populations. Secondly, the inclusion criteria were exclusively focused solely on patients with solitary nodules, which precluded the assessment of the success rate of localization for multiple nodules. Furthermore, temporal discrepancies exist between image acquisition and localization modeling, as the CT images used for modeling were not acquired simultaneously with the localization process, potentially introducing inconsistencies owing to minor positional changes. Additionally, there is a discrepancy in experience levels between individuals performing CT-guided biopsies and those conducting AR-guided operations, with the former possessing more extensive localization experience compared to the latter. Finally, the challenges in localization method were not blinded to the operator, although all measurements were conducted by individuals blinded to the grouping information. The rapid advancements in artificial intelligence (AI), exemplified by prominent models such as ChatGPT and Med-Gemini, have profound implications for the medical field^[29,30]^. In the future, the incorporation of AI into our device to assist with nodule localization and puncture path design may contribute to enhanced accuracy and safety in pulmonary nodule puncture procedures.

## Conclusion

The accuracy of AR-guided PTLP is not inferior to that of the CT-guided approach in localizing small lung nodules. Additionally, the AR approach shortened the procedure time and reduced the radiation exposure of patients.

## Data Availability

The dataset generated in this study is available upon reasonable request. Interested researchers can contact Dr Cheng to obtain the data.

## Research registration unique identifying number (UIN)

ClinicalTrials.gov identifier: NCT05715996.
